# Nicotinamide Adenine Dinucleotide Phosphate Oxidase 2 Expression and Effects of Alpha Lipoic Acid on Recovery in a Rat Model of Facial Nerve Injury

**DOI:** 10.3390/biomedicines10020291

**Published:** 2022-01-27

**Authors:** Myung Chul Yoo, In Yong Ryu, Jin Woo Choi, Jae Min Lee, Jae Yong Byun, Seung Geun Yeo

**Affiliations:** 1 Department of Physical Medicine & Rehabilitation, College of Medicine, Kyung Hee University, Seoul 02447, Korea; famousir@naver.com; 2 Department of Otorhinolaryngology, Head and Neck Surgery, College of Medicine, Kyung Hee University, Seoul 02447, Korea; nallert@naver.com (I.Y.R.); sunjaesa@hanmail.net (J.M.L.); otorhino512@naver.com (J.Y.B.); 3 Department of Pharmacy, College of Pharmacy, Kyung Hee University, Seoul 02447, Korea; jinwoo.ch@khu.ac.kr

**Keywords:** alpha lipoic acid, facial nerve injury, reactive oxygen species, NADPH oxidase 2

## Abstract

Background: NOX2 (nicotinamide adenine dinucleotide phosphate oxidase 2), which is upregulated by a variety of neurodegenerative factors, is neuroprotective and capable of reducing detrimental aspects of pathology following ischemic and traumatic brain injury, as well as in chronic neurodegenerative disorders. The purpose of this study was to investigate NOX2 expression and the degree of functional recovery following different types of facial nerve injury and assess the effects of antioxidant intervention on nerve regeneration. Methods: A total of 40 mature (6-week-old) male Sprague-Dawley (SD) rats were used. After inducing facial injury (compression injury or cutting injury), we randomized rats into four groups: A, crushing injury only; B, crushing injury with alpha lipoic acid (ALA); C, axotomy only; and D, axotomy with ALA. Recovery from facial nerve injury was evaluated 4 and 14 days after injury by performing behavioral assessments (observational scale of vibrissae movement, modified scale of eye closing and blinking reflex) and measuring changes in NOX2 experimental/control ratio in the injured (left, experimental) facial nerve relative to that in the uninjured (right, control) facial nerve. Results: A comparison between groups according to the type of injury showed a higher NOX2 expression ratio in the axotomy group than in the crushing group (*p* < 0.001). Regardless of injury type, both groups that received an injection of ALA exhibited a trend toward a higher NOX2 expression ratio, although this difference reached statistical significance only in the axotomy group (*p* < 0.001). In behavioral assessments, overall behavioral test scores were significantly higher in the crushing injury group immediately after the injury compared with that in the axotomy group. Additionally, in behavioral tests conducted 4 days after the crushing injury, the group injected with ALA showed better results than the group without injection of ALA (*p* = 0.031). Conclusions: Our study showed that NOX2 expression trended higher with facial nerve injury, exhibiting a significant increase with cutting-type injury. Furthermore, intraperitoneally injection with ALA may be an efficient strategy for accelerating peripheral facial nerve recovery after a crushing injury.

## 1. Introduction

Peripheral facial nerve injury can be caused by a variety of insults, including infection, inflammation, demyelination, trauma, iatrogenic injury, tumors, and compression [1,2]. The degree of facial nerve dysfunction after injury can vary from partial to complete paralysis depending on the patient, and the extent of recovery after treatment also varies. Facial paralysis is not a life-threatening condition, but if recovery is not complete, the appearance of the face is changed, creating discomfort in daily life, as well as mental stress. Therefore, recovery after facial nerve injury is crucial for the patient from both a social and mental health standpoint [3]. A number of studies have investigated treatment methods for improving functional recovery after facial nerve injury. Notable among these are ongoing studies attempting to improve recovery through the control of free radicals [4].

Generally, conservative treatment approaches, such as physical therapy and symptomatic treatment, are used to support nerve recovery from neuropraxia and axonotmesis [5]. Supplementary medications are also prescribed to support patient recovery; however, there is no global consensus on optimal treatment regimens. To date, several therapeutic agents, including steroids, non-steroidal anti-inflammatory drugs and vitamins, have been analyzed for their potential effects on the sciatic nerve regeneration process in a rat crush injury model [6,7,8]. Among the targets of therapeutic candidates are reactive oxygen species (ROS)—strongly reactive oxidizing compounds that include superoxide (O2•−), nitric oxide (NO•), hydrogen peroxide (H_2_O_2_), and peroxynitrite (ONOO•). Nicotinamide adenine dinucleotide phosphate oxidase 2 (NOX2), a member of the NADPH oxidase family of superoxide-producing enzyme complexes, has the primary function of generating free radicals. It is present in the outer membrane of neurons and is known to mediate the production of O2•− and H_2_O_2_ after nerve injury [9,10].

Alpha lipoic acid (ALA), a natural compound found in many prokaryotic and eukaryotic cell types, acts as a scavenger of various ROS. Literature reports indicate that ALA plays a crucial neuroprotective role by reducing free-radical–mediated oxidative stress after rat sciatic nerve crush injury [11]. Owing to its antioxidant activities, ALA has been widely studied as a treatment option for certain oxidative disorders of central and peripheral nervous systems, including stroke, spinal cord injury, neurodegenerative disorders, diabetic neuropathy, and ischemia-reperfusion injury [12,13,14]. However, evidence supporting ALA effects on the regeneration and functional recovery of injured facial nerves is lacking.

To test the potential role of ALA in peripheral nerve regeneration, we designed a facial nerve crush injury rat model and used it to investigate the relationship between free radicals and facial nerve recovery. Specifically, we sought to investigate NOX2 expression, and the degree of functional recovery following different types of facial nerve injury and assess the effects of antioxidant intervention on nerve regeneration. To this end, we used two different facial nerve injury paradigms—nerve crushing injury and axotomy—and monitored patterns of NOX2 expression in the peripheral facial nerve in relation to the extent of recovery after facial nerve injury and performed behavioral tests with and without ALA treatment.

## 2. Methods

### 2.1. Study Design and Preparation of the Nerve Injury Model

**Figure 1:**
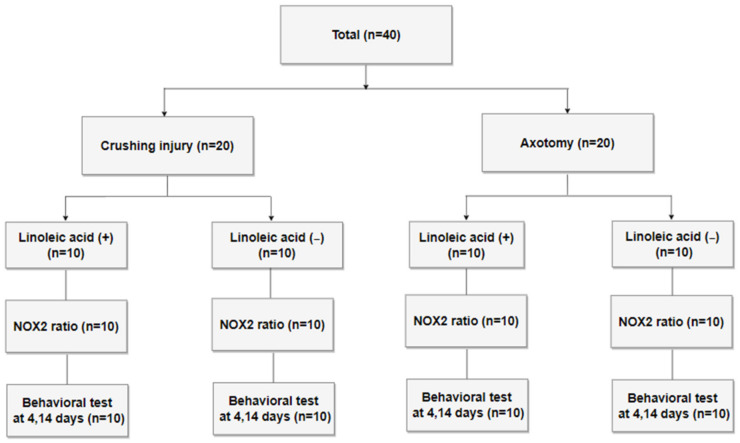
Study flow diagram.

**Figure 2:**
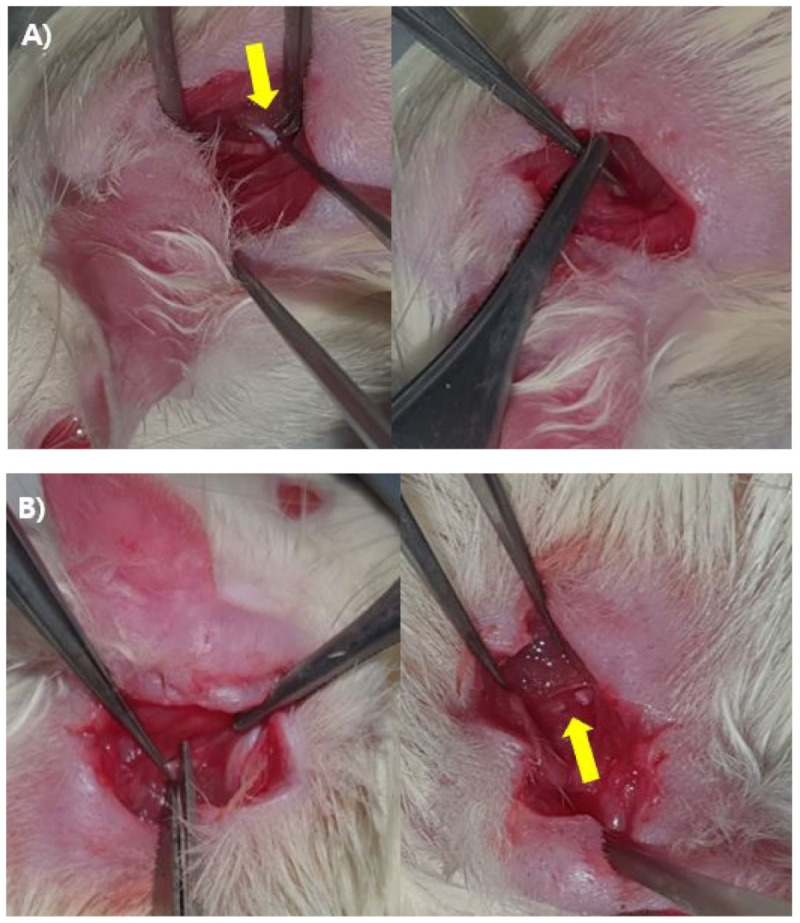
Experimental process for producing the left facial nerve injury model. A retroauricular incision in the skin and subcutaneous tissue was performed, after which dissection was done in planes. (**A**) Tendon border of the clavotrapezius muscle (yellow arrow), facial nerve trunk, and crushing injury to the proximal portion of facial nerve trunk. A crushing injury was produced by clamping the nerve for 30 s. (**B**) Axotomy on the proximal portion of the facial nerve trunk (yellow arrow).

Forty mature male Sprague–Dawley (SD) rats weighing less than 250 g were quarantined and acclimated for 1 week prior to experimentation. Animal breeding was performed in accordance with the Guidelines for the Use of Experimental Animals of the Institute of Medical Sciences, our university hospital. All rats received either a crushing injury (n = 20) or axotomy (n = 20) to the left facial nerve; the right facial nerve served as an uninjured control. Each group was further divided into an ALA-injected group and an untreated control group (10 rats/group) (Figure 1). For all surgeries, inhalation anesthesia was induced using 5% isoflurane (Forane solution; Joongeuk Pharmaceutical, Hwaseong, Korea) in 80% O_2_, and was maintained with 3% isoflurane. A left posterior incision was made in the anteromedial direction along the rear of the external auditory canal of anesthetized SD rats, after which the tendon of the clavotrapezius muscle was located and the main trunk of the facial nerve was exposed. Half of the rats received a compression or crushing injury in the proximal part of the main trunk of the facial nerve, created by clamping with two forceps for 30 s. For rats in the axotomy group, the proximal part of the main trunk of the facial nerve was completely cut and an anastomosis was made (Figure 2). Immediately after facial nerve injury, half of the rats in each injury-type group were intraperitoneally (i.p.) injected with 20 mg/kg/d of ALA (T5625; Sigma-Aldrich, Saint Louis, MO, USA) in 0.05 mL of 99% ethanol. The remaining rats (untreated vehicle controls) were intraperitoneally injected with 0.05 mL of 99% ethanol.

### 2.2. Behavioral Tests

The degree of nerve injury and facial nerve recovery was determined by assessing vibrissae movement using an observation-based scale and by evaluating corneal reflex using a modified scale of eye closing and blinking reflex. Both vibrissae movement and blinking reflex were quantified on 5-point scales, and average values were determined by two researchers based on videotaped measurements of behavioral tests. From the standpoint of vibrissae movement, complete functional recovery was defined as symmetrical anterior positioning and movement between the injured side and control side; this outcome, in which vibrissae on the injured side moved exactly the same as those on the opposite, non-injured side (normal movement, anterior), received a score of 5. Remaining outcomes were scored as follows: 4, normal vibrissae movement with a maintained posterior position; 3, tremor with posterior position; 2, light vibrissae tremor at the anterior position; and 1, normal vibrissae movement with a maintained posterior position [15]. Blink reflex was assessed using a modified 5-point scale of eye closing and blink reflex in which the ocular corneal area was stimulated with a soft cotton pad and the degree of eye blinking was measured [16]. Results were scored as follows: 5, complete eye closure with presence of a blinking reflex; 4, eye closure more than two thirds; 3, eye closure less than one half; 2, no eye closure with contraction of the orbicular muscle only; and 1, no movement [15]. Behavioral tests were performed three times. The first trial was performed on all 40 SD rats the day before the injury to the facial nerve to check for the presence of existing disorders. The second trial was performed after 4th day of facial nerve injury to confirm functional impairment. The third session was performed the 14th day before mice were sacrificed and facial nerves were collected.

### 2.3. Immunohistochemical Analysis of NOX2 Expression

NOX2 expression was measured in formalin-fixed right (control) and left (injured) facial nerves collected from anesthetized SD rats on days 4 and 14 after facial nerve injury. Formalin-fixed facial nerves were embedded in paraffin blocks, manufactured using a paraffin penetrator (ASP300S; LEICA, Germany), and then cut into 4-μm–thick sections and mounted on slides. Slides were incubated with a NOX2-specific rabbit primary antibody (1:200) for 1 h, followed by a 30-min incubation with an anti-rabbit secondary antibody (Envision). Slides were then washed with Tris-buffered saline containing 0.1% Tween-20 (TBST), and sites of NOX2 expression were detected by application of 3,3′-diaminobenzidine (DAB) together with Mayer’s hematoxylin staining to enhance contrast. Stained slides were scanned with a slide scanner (AXIO SCAN Z1; Carl Zeiss, Germany), and the area of NOX2 expression relative to the area of the main stem section of the facial nerve, expressed as a percentage, was calculated using software provided by the manufacturer (ZEN; Carl Zeiss). The difference in NOX2 expression between groups according to the type of injury and whether antioxidants were injected was determined from the ratio of NOX2 expression in injured left facial nerve to that of the uninjured right facial nerve of the same SD rat.

### 2.4. Statistical Analysis

Statistical analyses were performed using SPSS version 20.0 (IBM, Armonk, NY, USA). All values are presented as means ± standard error of the mean (SEM). NOX2 expression ratio data in the left injured facial nerve compared with that in the right control according to injury type were analyzed using three-way analysis of variance (ANOVA) with a multiple generalized linear model (GLM); an interaction test was additionally performed to confirm the interaction of injury type and ALA. The results of behavioral tests performed on the day of facial nerve collection were also analyzed using independent t-tests. In bar graphs and tables, *p*-values < 0.05 were considered statistically significant and “ns” indicates no significant difference.

## 3. Results

Using the experimental scheme depicted in Figure 1, we subjected Sprague-Dawley rats (n = 40) to either a crushing injury or cutting injury to the facial nerve (n = 20/group), as shown in Figure 2, and treated them with or without the antioxidant, ALA (i.p.). At two time points after injury (4 and 14 days), NOX2 expression ratios were monitored, and nerve recovery was assessed using behavioral tests.

**Figure 3:**
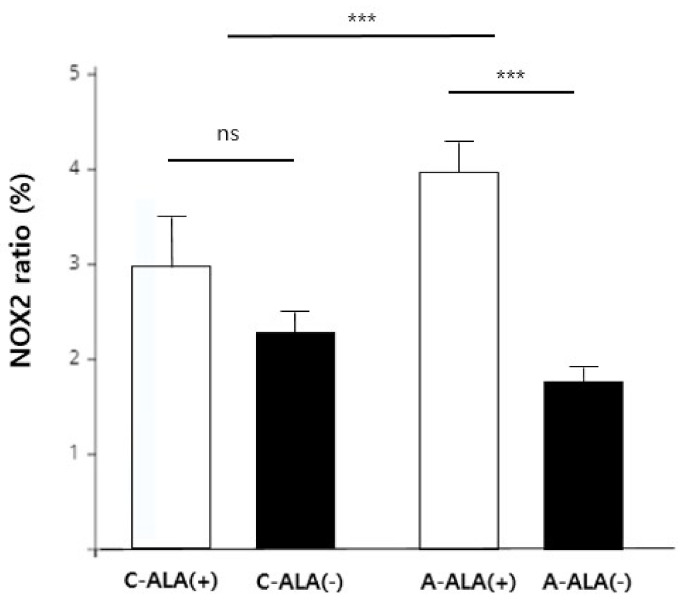
NOX2 expression ratio according to injury type and ALA treatment. NOX2 expression ratio between experimental groups, presented as bar graphs. C-ALA (+), crushing injury with ALA; C-ALA (−), crushing injury without ALA; A-ALA (+), axotomy with ALA; A-ALA (−), axotomy without ALA. Analyses performed using three-way ANOVA with a multiple generalized linear model, with additional interaction tests performed to confirm the interaction of injury type with ALA (adjusted time). *** *p* < 0.001; NS, not significant (*p* ≥ 0.05).

Analyses of NOX2 expression ratio data using three-way ANOVA with a multiple generalized linear model showed that only variables related to ALA treatment were statistically significant (Table 1, Figure 3). We further only found a significant interaction between injury type and ALA treatment on NOX2 experimental/control expression ratios. In the crushing injury group, NOX2 expression ratio was not significantly affected by injection of ALA (*p* > 0.05; Figure 3). However, the NOX2 experimental/control ratio was significantly higher (*p* < 0.001; Figure 3) in the axotomy group that received an injection of ALA compared with that in the group without ALA.

Behavioral tests showed that vibrissae movement and blink reflexes decreased in both crushing injury and axotomy models. On day 4 after injury, there was no significant difference in vibrissae movement or blink reflex between crushing injury and axotomy groups. However, on day 14 after injury, the vibrissae movement score in the crushing injury group was 1.95 ± 0.14, which was significantly higher than that in the axotomy group, with a score of 1.20 ± 0.13 (*p* < 0.001; Table 2). The blink reflex score was also higher in the crushing injury group than in the axotomy group. In contrast, the degree of facial paralysis was more severe in the axotomy group than in the crushing injury at the 14-day time point (*p* < 0.001; Table 2).

Notably, application of ALA tended to improve behavioral test scores in the crushing injury group on days 4. In particular, the blink reflex score on day 4 was 1.90 ± 0.24 in the ALA injection group, which was significantly higher than the average score of 1.20 ± 0.20 in the non-injection group (*p* = 0.031; Table 2). However, scores of behavioral tests in the axotomy group were the same or lower with ALA injection on days 4 and 14 compared with the non-injection group.

## 4. Discussion

Free radicals, generated by virtue of the electron-accepting property of oxygen, are highly reactive oxidative chemical molecules, formed as natural byproducts of the normal aerobic metabolism of oxygen, that play important roles in cellular signaling and homeostasis [17,18]. Free radicals contribute to tissue damage and remodeling after injury through inflammatory reactions. In addition, inhibition of free radicals through administration of antioxidants after nerve injury can prevent further tissue injury and thus aid in nerve recovery [4,11]. Although active oxygen can be directly measured using electron spin resonance (ESR), which detects unpaired electrons, it is rapidly lost through redox reactions in tissues owing to the high reactivity of free radicals. Therefore, the presence of active oxygen in tissues and cells is difficult to measure, and the accuracy of quantitative measurements is poor [19,20].

Numerous studies have sought to establish a stable detection index that represents the active oxygen level. One representative marker is NOX2, which was used in the current study. NOX is a cell membrane protein that enzymatically converts molecular oxygen (O_2_) into active oxygen and superoxide (O2•−), and superoxide to hydrogen peroxide (H_2_O_2_). When tissue and cell damage occur, intracellular synthesis of ROS is increased by inflammatory cytokines such as TNF-α and IL-1. The NOX family is known to include five NADPH oxidase members (NOX1-5) and two dual oxidase members (DUOX1 and DUOX2). NOX2 is expressed at high levels in phagocytes found in inflammatory tissues and is moderately expressed in nerve cells. NOX2, the focus of the current study, has been previously used as an indicator of changes in free radicals in facial nerve injury [9]. Because IL-1, TNF-α, and TGF-β are increased during peripheral nerve injury in rats [21,22], it is thought that the higher NOX2 levels in the facial nerve in the injured (left) side compared with the uninjured (right) side might reflect induction of NOX2 expression by these inflammatory cytokines [9,23,24].

In this study, antioxidants were administered to assess differences in NOX2 expression and recovery according to changes in ROS and to test the therapeutic effects of antioxidants. Well-known antioxidants include the physiologically produced compounds glutathione, superoxide dismutase and ALA, as well as vitamin C and vitamin E, which are consumed as part of human diets. ALA is approved for use as a therapeutic agent in diabetic neuropathy and has been used in several previously published articles to study the relationship between antioxidants and recovery after nerve injury [25].

ALA acts directly on NOX2 to inhibit the production of O2•− and H_2_O_2_, and neutralizes generated free radicals, inhibiting the cell damage caused by them [26]. In previous studies using related rat models, ALA was infused at doses ranging from 10 to 50 mg/kg/d and was shown to cause death at the highest dose. In addition, long-term (3 weeks) dosing experiments showed that, at a dose of 20 mg/kg/d, ALA caused no side effects [27,28]. Thus, this dose of ALA was used for intraperitoneal injection in the current series of experiments, and ethanol was used as a solvent for ALA. One of the most commonly used metrics for reporting the toxicity of chemicals is the median lethal dose (LD_50_), which represents the dose that causes 50% mortality. The LD_50_ of ethanol is between 5.10 and 6.71 g/kg, depending on the age of the rat, and the 50% effective dose 50% (ED_50_) is reported to be as low as 239.4 mg/kg. The amount of ethanol used as a solvent in our experiments (0.05 mL) corresponds to an ethanol dose of 157.8 mg/kg for 250 g rats, which is significantly lower than the LD_50_ or ED_50_ [29,30] and thus can be considered a safe dose. The same amount of 99% ethanol (0.05 mL) was injected the same number of times in both vehicle control and ALA injection groups. Although direct injury from intraperitoneal injection of 99% ethanol is rare, it has been reported to cause temporary fibrosis of the abdominal wall cavity [31]. Therefore, in this study, 0.05 mL of 99% ethanol was injected into control group animals that were not injected with ALA to achieve the same conditions as in the ALA-injection group.

As shown in Table 2, there was no difference in behavioral test responses between injury types on the 4th day after nerve injury. However, overall behavioral test scores in the crushing injury group showed greater improvement on day 14 relative to that immediately after the injury than did the axotomy group, indicating that the degree of recovery is faster with less axonal loss or damage. Additionally, rats in the crushing injury group injected with ALA showed better results in behavioral response tests (eye closing) conducted on the 4th day after the injury than the group without injection of ALA, consistent with previous results showing recovery faster after nerve crushing injury with application of ALA [4,11]. The absence of a significant difference with ALA on day 14 in the crushing injury group may reflect the fact that significant recovery is observed through peripheral nerve regeneration at this later time point regardless of active oxygen suppression [32]. In the case of axotomy, the degree of recovery on the 4th day was poor with or without ALA and failed to show improvement on the 14th day, similarly indicating that more time is required for recovery of total axonal loss.

The difference in behavioral test results between crushing injury and axotomy groups can be explained by the differences in recovery process that, in turn, reflect the severity of the injury and continuity of the nerve. After a nerve crushing injury, axons in the nerve cell body retain their axonal continuity; in particular, Schwann cells surrounding the nerve cell body are highly likely to survive. Even if it is assumed that a crushing injury causes excessive axonal damage and destroys nerve continuity, the basal plate of the neural tube, a tough fibrous membrane surrounding the axon, is maintained. Therefore, new axons can quickly regrow and reconnect along the basal plate of the neural tube, thereby rapidly achieving complete functional recovery. In other words, in the crushing injury group, where neuronal continuity is highly likely to be maintained, the prevention of additional nerve damage mechanisms due to the inflammatory actions of ROS is important. This is evidenced by the beneficial effects of inhibiting the action of free radicals by injected ALA, which helped to restore facial nerve function in SD rats. However, in the axotomy group, both the axon and the basal plate of the neural tube are destroyed by severing the nerve; thus, the continuity between the proximal nerve cell body and the end of the distal axon is completely lost. In this scenario, nerve regeneration proceeds through formation of a new tissue “bridge” between the broken structures that reconnects proximal and distal stumps. To span this gap, the axon and basal plate of the neural tube must be reconstructed from the beginning at the proximal part of the nerve cell body [33]. At the same time, ROS promote dieback and degeneration of the end of the axon after axonal injury, thereby aiding the initial axonal regeneration and functional recovery of the nerve. Since active oxygen, which is required for axonal regeneration and functional recovery of transected nerves, is neutralized by ALA, it appears that antioxidant treatment is not thought to help facial nerve regeneration in this setting [34]. Furthermore, ROS signaling is required to drive both peripheral and central nervous system axon regeneration in response to regulatory sciatic nerve injury [34]. ROS signaling is initiated by exogenous NOX2 delivered through extracellular vesicles derived from cytokine-recruited inflammatory macrophages [35].

The NOX2 experimental/control expression ratio differed depending on the type of injury. After a crushing injury, the NOX2 experimental/control expression ratio in the ALA-injection group was higher than that in the non-injection group. Similarly, since a high level of ROS is required in the axotomy group, based on the recovery mechanism described above, ALA administration is thought to further promote the expression of NOX2 to meet the reactive oxygen demand. In the crushing injury group, there was no significant difference in the NOX2 expression ratio after injury between groups with and without ALA injection. This lack of a difference is likely attributable to the fact that the need for active oxygen in the recovery mechanism is lower in the crushing injury group than in the axotomy group. In particular, rats in the axotomy group treated with ALA exhibited a significant increase in NOX2 compared with the group without ALA administration. The finding of high NOX2 despite diminished active oxygen in tissue through administration of ALA may be explained as follows: (1) Soon after a lesion, injured axons experience inflammation reflecting the extremely oxidative environment; this contributes to the initial axonal collapse and retraction, which occurs via ROS-dependent oxidation [36]. (2) Axonal dieback and degeneration releases ROS, which affect axonal signaling, potentially supporting the regenerative program after nerve injury and a conditioning lesion. (3) Following the administration of ALA, which results in a ROS-scavenging effect, cells produce more ROS through NOX2 to restore their functional recovery.

Prior studies on the role of NOX2 in various animal models and the mechanism of regeneration for peripheral nerve injury have been reported. In response to peripheral nerve injury, NOX2-positive macrophages were recruited to dorsal root ganglia, and ROS production was increased in a NOX2-dependent manner [37]. ALA induces the moderate production of ROS, which in this content serve as signaling molecules and lead to the activation of ERK. The activation of this pathway results in neurite outgrowth [38]. In addition, Krämer-Albers have shown that injured nerves recruit macrophages that release extracellular vesicles carrying the ROS-producing NOX2 complex. NOX2 is internalized at the site of injury and undergoes retrograde transport in endosomes. NOX2–ROS signalling mediates elevation of pAkt levels and oxidation of PTEN, leading to axon outgrowth after injury [35]. The reason for the faster recovery on the 4th day in the crushing group treated with ALA is similar: administration of ALA increases ROS, which acts as a signaling molecule, reflecting increased formation of ROS-producing NOX2 complexes [35]. The implication is that, by promoting neural outgrowth, ALA leads to relatively rapid recovery in behavioral outcomes. However, NOX2 expression was significantly increased in the axotomy group treated with ALA compared with ALA-untreated controls. Because of axonal dieback and degeneration after axotomy, nerve regeneration takes longer; therefore, it may be more difficult for ALA to produce meaningful results in the axotomy group than in with the crushing group.

The present study had several limitations. First, this study did not include a control group of sham-operated rats to exclude the effects of peripheral tissue injury. The expression of NOX2 and other inflammatory cytokines may also have been influenced by facial muscle incisions without facial nerve axotomy. Second, this study evaluated only NOX2 expression, not the expression of proinflammatory cytokines, and did not include immunohistochemical assays. Inclusion of these assays may provide strong evidence about the association between inflammatory cytokines and pathological information in response to ALA administration. Additional studies are needed to confirm our results, showing that ALA treatment is effective in a rat model of facial nerve injury.

## 5. Conclusions

The current study is to provide experimental evidence demonstrating that injection with ALA can promote nerve regeneration in a model of rat crushing injury. Our study also showed significantly higher NOX2 expression following facial nerve injury—in particular, a nerve cutting injury. Intraperitoneally injection with ALA may be an efficient strategy for accelerating peripheral facial nerve recovery after a crushing injury.

## Figures and Tables

**Table 1:** NOX2 expression ratios, evaluated using three-way ANOVA with a multiple generalized linear model.

Variable	β Estimate	95% CI		*p*-Value
Injury type				
Crushing injury	0.21	−0.45	0.88	0.520
Axotomy	0.00			
Time after injury				
Day 4	−0.60	−1.27	0.07	0.076
Day 14	0.00			
ALA treatment				
ALA (−)	−1.57	−2.24	−0.90	<0.001 *
ALA (+)	0.00			

Data are presented as differences (95% confidence intervals [CI]); * *p* < 0.001. ALA, alpha lipoic acid.

**Table 2:** Comparison of behavioral test scoring between the injury type, time and ALA treatment.

Time after Injury	Vibrissae Eye Closing					
	Crushing	Axotomy	*p*	Crushing	Axotomy	*p*
Day 4	1.30 ± 0.14	1.20 ± 0.13	0.586	1.60 ± 0.17	1.20 ± 0.15	0.274
Day 14	1.95 ± 0.14	1.20 ± 0.13	<0.001 *	2.30 ± 0.17	1.30 ± 0.15	<0.001 *
Time after injury	Vibrissae Eye closing					
	C-ALA (+)	C-ALA (−)	*p*	C-ALA (+)	C-ALA (−)	*p*
Day 4	1.30 ± 0.20	1.20 ± 0.20	0.374	1.90 ± 0.24	1.20 ± 0.20	0.031 †
Day 14	2.00 ± 0.20	1.90 ± 0.20	0.478	2.20 ± 0.25	2.00 ± 0.20	0.374
Time after injury	Vibrissae Eye closing					
	A-ALA (+)	A-ALA (−)	*p*	A-ALA (+)	A-ALA (−)	*p*
Day 4	1.50 ± 0.15	1.70 ± 0.15	0.524	1.50 ± 0.15	1.50 ± 0.15	1.000
Day 14	1.40 ± 0.15	1.60 ± 0.15	0.580	1.30 ± 0.15	1.40 ± 0.15	0.450

Data are presented as number (mean ± SD) and *p*-value. * *p* < 0.001, † *p* < 0.05. Vibrissae, vibrissae movement; Eye closing, eye closing and blinking reflex; C-ALA (+), crushing injury with ALA; C-ALA (−), crushing injury without ALA; A-ALA (+), axotomy with ALA; A-ALA (−), axotomy without ALA.

## Data Availability

Not applicable.
